# Statistical Methodologies for the Optimization of Lipase and Biosurfactant by *Ochrobactrum intermedium* Strain MZV101 in an Identical Medium for Detergent Applications

**DOI:** 10.3390/molecules22091460

**Published:** 2017-09-11

**Authors:** Gholamhossein Ebrahimipour, Hossein Sadeghi, Mina Zarinviarsagh

**Affiliations:** Department of Microbiology and Microbial Biotechnology, Faculty of Biological Sciences and Technology, University of Shahid-Beheshty, Tehran 1983963113, Iran; g-ebrahimi@sbu.ac.ir (G.E.); sadeghi.hsn88@gmail.com (H.S.)

**Keywords:** *Ochrobactrum intermedium* strain MZV101, lipase, biosurfactant, detergent

## Abstract

The Plackett–Burman design and the Box–Behnken design, statistical methodologies, were employed for the optimization lipase and biosurfactant production by *Ochrobactrum intermedium* strain MZV101 in an identical broth medium for detergent applications. Environmental factor pH determined to be most mutual significant variables on production. A high concentration of molasses at high temperature and pH has a negative effect on lipase and biosurfactant production by *O. intermedium* strain MZV101. The chosen mathematical method of medium optimization was sufficient for improving the industrial production of lipase and biosurfactant by bacteria, which were respectively increased 3.46- and 1.89-fold. The duration of maximum production became 24 h shorter, so it was fast and cost-saving. In conclusion, lipase and biosurfactant production by *O. intermedium* strain MZV101 in an identical culture medium at pH 10.5–11 and 50–60 °C, with 1 g/L of molasses, seemed to be economical, fast, and effective for the enhancement of yield percentage for use in detergent applications.

## 1. Introduction

Lipases (EC 3.1. 1.3) are enzymes that hydrolyze fatty acyl ester bonds of acylglycerols at the interface between lipid and water [1]. Biosurfactants have diverse structural components that consist of hydrophilic (polar) and hydrophobic (non-polar) groups and are categorized by their chemical composition and microbial origin [2,3]. Biosurfactants and lipases are both polymers produced by bacteria, fungi, and yeast [4]. Biosurfactants are used as an important component in commercial laundry detergent due to their environmentally friendly characteristics, i.e. biodegradability and low toxicity [5,6]. Biosurfactants, along with other enzymes and detergent elements, could enhance stain elimination efficiency [7]. Substances that are stable and functional at a high pH level and temperature and that are adaptable under production conditions are required for detergent formulations [8,9].

Despite all lipase and biosurfactant benefits, the high cost of medium material, slow processing, and low yield are the main economic issues in detergency manufactories. Bacteria production with inexpensive waste argo and industrial supplements, such as whey cheese, molasses, and cheap vegetable oils, could solve these manufacturing problems [10].

The biosurfactant and lipase synthesis of different microorganisms may be affected by environmental factors, such as pH, temperature, and agitation speed, that either increase or inhibit its productivity. In order to enhance production, the use of suitable nutrition and environmental parameters is essential [1,11]. The same conditions cannot be suitable for every kind of microorganism [12].

In addition, production in large industrial quantities demands the optimum achievement of various parameters [7]. The production of biosurfactants in an identical medium with other enzymes used in detergent applications will reduce cost and save time [13].

The Plackett–Burman design is a screening design used for distinguishing vital variables among other variables; so far, only the main effects have been evaluated [14,15]. Employing classical methods of optimization one factor at a time requires a large number of experiments. Further, the classic method does not define the relation between factors and effects of each factor [16]. Additionally, the Box–Behnken design depicts the interaction between independent variables and predicts the bacteria response [17]. Hence, new models are an efficient method of evaluating the best optimum medium condition in order to achieve the highest yield in an economical manner [11,15].

The aim of the present study was to investigate the increase rate of lipase and biosurfactant production by *O. intermedium* strain MZV101 in an identical medium in response to different factors by using statistical analysis for detergent applications. In the first step, we used the Plackett–Burman design to evaluate the effect of the most significant mutual factors of the production rate bacteria. Response surface methodology was then used to further investigate the mutual significance factor of lipase and biosurfactant production by *O. intermedium* strain MZV101.

## 2. Results

### 2.1. Plackett–Burman Design

In this paper, the Plackett–Burman design was employed to evaluate the most significant factor between 11 independent variables: environmental factors, the nitrogen source, the oily carbon source, and the media components in the production of biosurfactant and lipase by *O. intermedium* strain MZV101 using an identical basal medium. The 11 uncoded variables used in the Plackett–Burman design with their response are presented in Table 1.

**Figure 1:**
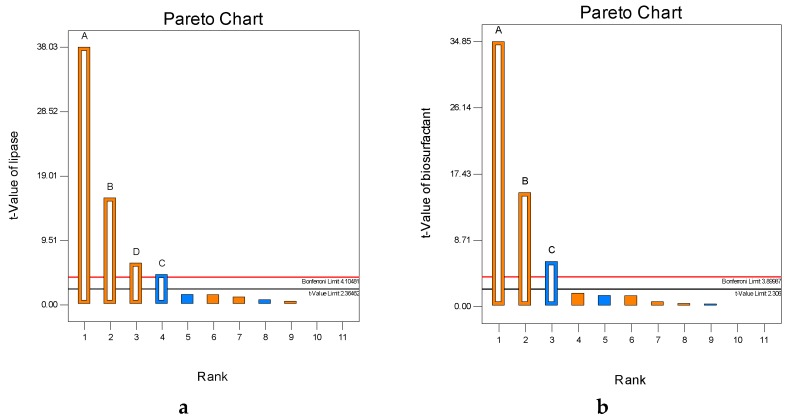
Pareto charts (**a**) lipase and (**b**) biosurfactant production by *O. intermedium* strain MZV101. This chart based on the observations of the Plackett–Burman experimental design for evaluating different variables on lipase and biosurfactant production by the above-mentioned strain, where A is pH, B is temperature, C is molasses in both figures, and D is MgSO₄. The orange color indicates a positive effect, while the blue color indicates a negative effect on the lipase and biosurfactant.

The Plackett–Burman design results show that temperature, pH, MgSO₄, and molasses were determined to be the most effective factors in lipase production, which is confirmed by the Pareto chart as shown in Figure 1a. The results indicated that temperature, pH, and molasses in the basal medium significantly affected biosurfactant production, and these results are confirmed by the Pareto chart as shown in Figure 1b. The Plackett–Burman design suggested that pH, temperature, and molasses were significant mutual factors on biosurfactant and lipase production.

The main effect (*F*-value, *p*-value) and co-efficient of each variable were evaluated in terms of biosurfactant and lipase production (Table 2).

### 2.2 Response Surface Methodology

In the next step, for further optimization, the Box–Behnken design was used. However, divalent cations such as MgSO₄, along with environmental factors and nutritional parameters, have an important role in the enzyme production in detergent applications [18]. We continued our experiment by choosing mutually significant independence parameters between lipase and biosurfactant for optimization in the same medium and condition. Therefore, three mutual parameters-pH (8, 10 and 12), temperature (20, 40 and 60 °C), and (1, 2 and 3 g/L) molasses were chosen. The Box–Behnken experimental design consisted of 30 runs with five levels of three variables, and experiments were carried out in a random order. The Box–Behnken design and their results are presented in Table 3. Our experimental results were close to the predicted values.

A response with a *p*-value < 0.05 was considered significant in lipase and biosurfactant production. The statistical analysis results imply that the model *F*-value of 54.08 for lipase is significant. The statistical analysis results imply that the model *F*-value of 250.58 for biosurfactant is significant.

The quadratic model represents lipase enzyme activity Y (U/mL) and emulsification index Y (%) as a function of pH (X₁), temperature (X₂), and molasses (X₃). A *p*-value < 0.05 indicated the significance of each coefficient. The production of lipase enzyme Y (U/mL) was predicted by the following model equation:

(1) $$Y\;\left(U/\mathrm{mL}\right)\;=5.52+\;1.50{\;X}^{1}+\;0.85{\;X}^{2}-\;0.62{\;X}^{3}+\;0.19{\;X}^{1}X^{2}-\;2.81{{\;X}^{1}}^{2}+\;0.026{{\;X}^{2}}^{2}-0.037\;{X_{3}}^{2}$$

Biosurfactant production Y (%) was predicted as shown in the equation below:

(2) $$Y\;\left(\%\right)=83.24+\;12.75{\;X}^{1}+\;4.34{\;X}^{2}+\;1.35\;X₃-\;32.02\;{X_{1}}^{2}\;.$$

The results of the ANOVA for response surface quadratic model are illustrated in Table 4. The model *p*-value of <0.0001 is significant for the lipase enzyme model. The model *F*-value of 5284.42 indicates that the model is significant. The lack-of-fit for lipase (*F*-value 3.22) was not significant, which implies that the models have a correlation between the independent variables and the responses. The correlation coefficient (R²) value at 95% confidence level was observed to be 0.9998. The value of the adjusted R-squared was 0.9995. The value of the predicted R-squared was 0.9994. The Adeq Precision of lipase was 301.557. Based on the Adeq Precision results, the ratio was considered to be desirable. According to the results of ANOVA for response surface quadratic model, the pH with the highest *F*-value (lipase) of 15939.05 has the greatest effect on lipase production (Table 4). As shown in Table 4, the temperature with an *F*-value (lipase) of 4966.13 after pH, have a high and a positive effect on lipase production by strain MZV101.

The model *F*-value of 6371.84 for biosurfactant indicates that the model is significant. The lack-of-fit (*F*-value 0.07899) was not significant. The correlation coefficient (R²) value at 95% confidence level was observed to be 0.9997. The value of the adjusted R-squared was 0.9997. The value of the predicted R-squared was 0.9995 is a reasonable agreement with the adjusted R-squared. The Adeq Precision of biosurfactant was 211.874. Based on Adeq Precision results, the ratio was considered to be desirable. According to the results of the ANOVA for the response surface quadratic model, the pH with the highest *F*-value (biosurfactant) of 14069.85 has the greatest effect on biosurfactant production (Table 4). As shown in Table 4, the temperature with an *F*-value (biosurfactant) of 1632.34 after pH has a great and positive effect on biosurfactant production by strain MZV101. From the results of the Box-Behnken design, and the ANOVA table, we can conclude that pH was the most effective factor among all parameters.

**Figure 2:**
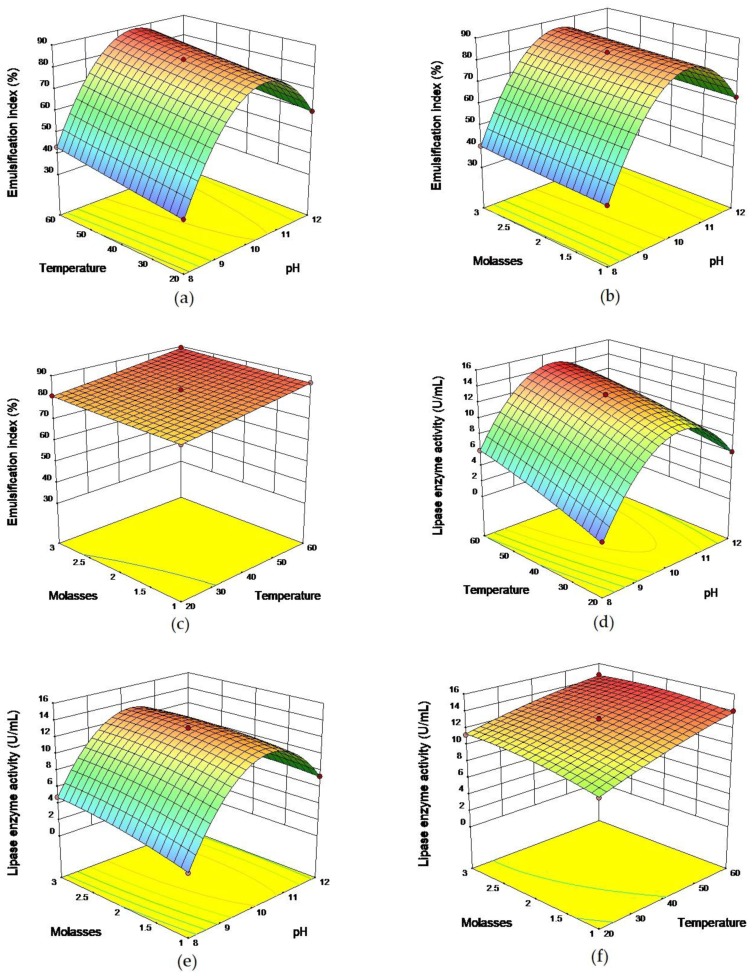
Three-dimensional (3D) response surface plots and their interaction effect between variables for lipase and biosurfactant activity by *O. intermedium* strain MZV101. (**a**,**d**) Interactions between pH and temperature, (**b**,**e**) interactions between molasses and pH, and (**c**,**f**) interactions between molasses and temperature in basal media with 10 g/L of olive oil with lipases and biosurfactants stimulated by the above-mentioned bacteria.

The three-dimensional (3D) response surface plots shown in Figure 2 indicate the interactive effects of pH, temperature, and molasses.

Interaction between pH and temperature in the biosurfactant is shown in Figure 2a. The highest emulsification index value for *O. intermedium* strain MZV101 was observed at pH 10.5 and 60 °C (Figure 2a). It can be seen that biosurfactant production values were directly affected by temperature and pH. Most biosurfactant production by bacteria is affected by environmental factors such as temperature and pH. Therefore, it is important to optimize environmental factors pH and temperature to obtain a maximum yield in bioprocesses. Zinjarde and Pant Pant et al. reported the best biosurfactant production by *Yarrowia lipolytica* NCIM 3589 at 30 °C in the natural pH 8 of sea water , which is in contrast to our results [19].

Interaction between pH and temperature on lipase production is illustrated in Figure 2d. The highest lipase activity for *O. intermedium* strain MZV101 was attained at pH 10.5 and 55–60 °C. However, as illustrated in Figure 2d, lipase production values were significantly influenced by temperature and pH, and activity increased with increases in temperatures and pH. Bisht et al. investigated the optimization of alkaline lipase production from *Pseudomonas aeruginosa* using the Plackett–Burman design. In their description of the interaction between temperature and pH, they reported that the effect of a high temperature and pH on the amount of lipase production was negative. The highest lipase activity was observed at 35 °C and pH 8.5 [20]. In contrast to our results, Khoramnia et al. showed that temperature plays an important role in lipase production from *Acinetobacter* sp., while pH had no significant effect in the optimization process [21].

The majority of microorganisms are produced the lipase enzyme at pH 7–10 [22]. The optimal temperature varies for every microorganism strain, and this must be investigated. The optimum temperature for *O. intermedium* strains reported to be 20–37 °C and optimum pH are 7–7.3 [23]. Noparat et al. have reported the optimum temperature and pH for biosurfactant production from *Ochrobactrum* *anthropi* 2/3 in a nutrient broth medium at 30 °C and pH 7 were achieved [24]. Mishra et al. reported the optimal temperature and pH for biosurfactant and lipase production by *Ochrobactrum intermedium* P2 in a mineral salt medium were respectively 37 °C and pH 7 [22]. From these results, we can state that no report is available in the literature regarding the same strain *O. intermedium* producing lipases and biosurfactants with an optimum pH of 10.5–11 and a temperature of 55–60 °C.

Specific biochemical characterization is required for each industrial application [25]. Detergent formulations along with enzymes and other substances often function at 30–60 °C and alkaliphilic pH levels 9–12, and compatibility with other compounds have been described [26,27]. A functional lipase enzyme at a wide range of temperatures and pH levels for detergent applications is an ideal choice. In general, laundering under an alkaline condition is preferred. [28]. Under an alkaline condition, fatty stains are removed more easily from cloth, but an alkaline solution is unable to soponify stains, so in this case stains are not removed from the textile material. By adding a lipase enzyme, oily stains can be easily removed from a given fabric [23,29]. We found lipase from *O. intermedium* strain MZV101 as an additive component in detergent form in high temperature and pH. Cherif et al. found that the lipase from the Staphylococcus sp. strain ESW is an ideal choice in detergent formulations, with optimum activity at pH 12 and 60 °C. Additionally, Bora et al. observed that the lipase from *Bacillus* sp. DH4 as a detergent additive has optimum activity at pH 9 and 60 °C [29]. These studies are in good agreement with our results [30]. In contrast to our results, Hemachander et al. have reported that lipase from *Ralstonia pickettii* as an additive component in detergent formulations have an optimum activity at lower temperature 37 °C and pH 6.5 [31].

Figure 2b shows the interaction between molasses and pH in biosurfactant production.

The maximum emulification index values were observed with 1–3 g/L of molasses at pH 11–12. pH as an environmental factor plays an important role in biosurfactant production, while molasses with different concentrations have no effect on biosurfactant production. These findings are in accordance with those of Shavandi et al. who observed that the *Serratia marcescens* NSK-1 strain capacity was very sensitive to pH [32]. Korayem et al. have reported that the highest biosurfactant production E24 34.1% from *Streptomyces* sp. was obtained using the Plackett–Burman design in a medium containing 20 g/L of molasses as a carbon source at 30 °C and pH 8 for 72 h [14]. Our study showed an inferior value for biosurfactant production.

Most maximum biosurfactant emulsification index values from *Ochrobactrum* strains were reported to be in low pH and temperature. For instance, Ferhat et al. have declared that biosurfactant production by *Ochrobactrum* sp. 1C C1in a medium containing 2% hexadecane as the carbon source increased in lower pH between 4 and 7 (E24 average of 95%) at 37 °C and at 200 rpm for 48 h was obtained [33]. Bhattacharya et al. observed that the highest emulsification index values E24 69.42 ± 0.32% were from *Ochrobactrum* sp. C1 using the Box-Behnken design in a medium containing 4.6% waste engine oil at pH 7.3 and 36.4 °C [34]. Lipases and biosurfactants by *O. intermedium* strain MZV101 with suitable stability at high temperature and pH are therefore promising for use in detergent applications.

Figure 2e depicts the interaction between molasses and pH on lipase production. The maximum lipase activity was about 14.5 U/mL at pH 10.5 with 1 g/L of molasses. Various concentrations of molasses have no significant effect on lipase production. In contrast to our results, Potumarthi et al. demonstrated a maximum lipase activity of 72 U/mL from *R. mucilaginosa* MTCC 8737 using 1% molasses in pH 7 and 25 °C after 96 h [35].

pH is a factor that can affect protein structure and consequently the enzyme activity. Lipases produced by bacteria have been reported to show optimum activity at pH 5–9 [36]. Thus, enzyme activity and stability at alkaline pH is a crucial factor in detergent formulations [37]. Chen et alobserved that alkaline lipase from *Achetobacter radioresistens* for detergent applications in a medium containing 0.1% olive oil and 1.5% *n*-hexadecane had an optimum pH of 10.0 at 30 °C [38].

Interaction between molasses and temperature is shown in Figure 2c for biosurfactant production. At high temperatures and high concentration molasses, emulsification index values decreased. Biosurfactant production from *O. intermedium* strain MZV101 was obtained in a maximal amount from 1 g/L of molasses at high temperature, while Joshi et al. observed maximum biosurfactant production from *Bacillus subtilis* 20B, by using 5–7% molasses as a carbon source at 55 °C and pH 8 [39].

Figure 2f illustrates the interaction between molasses and temperature on lipase production. The highest yield of lipase enzyme activity (14.01 U/mL) was obtained at 55 °C with 1 g/L of molasses. These results suggest that high concentrations of molasses have a reverse relationship with the production of lipase production by *O. intermedium* strain MZV101 in this medium. Comparable to our observed in this study, Rathi et al. reported on the alkaline lipase production from *Burkholderia cepacia* RGP-10 for detergent formulations in a medium containing 1% oil at 50 °C, pH 7, and 250 rpm, which was mainly influenced by 1% glucose and 0.6 mM Mg^2+^ in the presence 0.4 mM Ca^2+^, and lipase production increased more than 3-fold [23].

### 2.3 Validation of the Model

**Figure 3:**
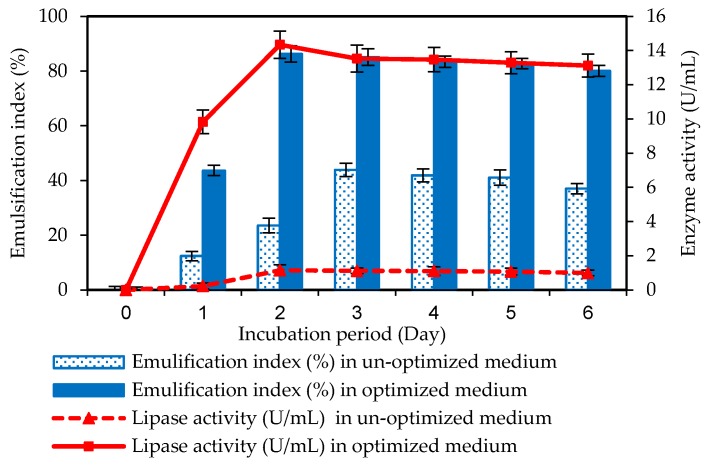
Lipase enzyme and biosurfactant production in un-optimized and optimized basal medium by *O. intermedium* strain MZV101. Results were represented as mean ± S.D. All tests are repeated in at least three independent experiments. Error bars indicated standard deviations as shown in this figure.

To confirm validation of the planned model, lipase and biosurfactant production by *O. intermedium* strain MZV101 was studied in the presence of culture-optimized and un-optimized media (Figure 3).

The isolated bacteria was cultured in an un-optimized basal medium containing 1 g/L of yeast extract and 10 g/L of olive oil at 60 °C and pH 10 with a 120 rpm agitation speed for 6 days. Gutarra et al. reported the highest lipase activity of 20 U from *Penicillium simplicissimum* was obtained by utilizing olive oil or molasses as a single supplement at pH 7 and 30 °C [40].

In an optimized basal medium, we added 1 g/L of molasses and adjusted the pH to 11, and other conditions were the same as the un-optimized medium.

The maximum lipase and biosurfactant production was achieved both in an un-optimized medium after 72 h and in an optimized medium after 48 h after inoculation of *O. intermedium* strain MZV101. From the results, we can claim that production and growth duration was 24 h shorter. Mishra also reported maximum lipase production from *O.* *intermedium* strain P2 was observed after 48 h of incubation [22].

The production of lipase in an un-optimized medium was 4.19 U/mL, compared to 14.53 U/mL in the optimized media. This result confirmed that a 3.46-fold increase in lipase production was observed in the optimized medium. Therefore, we demonstrate here that a low concentration of molasses along with oil can positively affect lipase production at a high pH and temperature. Our findings are in contrast to other studies. For instance, Açıkel et al. used the Box–Behnken design to optimize the lipase from *Rhizopus* *delemar*. The optimum fermentation medium composition and condition was 0.53 g/L of yeast extract, 1.32 g/L of molasses, and 10 g/L of sunflower oil at a low pH of 6 and at 30 °C [41].

Papagora et al. reported that lipase production from *Debaryomyces hansenii* were increased approximately 2.5-fold using response surface methodology in a medium containing 13.1 g/L of glucose and 19 g/L of olive oil at 30 °C and pH 6.4 with 150 rpm for 72 h [42]. Kai and Peisheng reported that the optimal lipase activity of 11.49 U from *Thalassospira permensis* M35-15 using the Box–Behnken design and response surface methodology in optimum medium containing 5.15 g/L of glucose, 11.74 g/L of peptone, 6.74 g/L of yeast powder, and 22.90 g/L of olive oil emulsifier at pH 7 and 28 °Ϲ increased 1.85-fold [43]. Our results, compared to this report, shows a three-fold higher yield in lipase production, with less material utilization.

García-Silvera et al. investigated extracellular lipase production from wild-type *Serratia marcescens* and three mutant strains for detergent formulation and biodiesel production using a 2² factorial design. Lipase production increased to 124 U/mL, about 1.55-fold higher than the initial ratio. The optimum medium composition and conditions were 4% soybean oil and 0.05% Triton X-100 at 50 °C and pH 8 with 200 rpm after 48 h of incubation [44]. Our study shows an inferior value for lipase production by *O. intermedium* strain MZV101.

An emulsification index of 45.54% biosurfactant in the un-optimized medium, as compared to 86.23% biosurfactant in the optimized medium, was found. The results show a 1.89-fold increase in biosurfactant production. According to our results, we can declare that a low concentration of a hydrophobic carbon source (olive oil, corn oil, etc.) and a low concentration of a hydrophilic carbon source (molasses, glucose, etc.) at a high pH and temperature can enhance the amount of biosurfactant production by *O. intermedium* strain MZV101. Others studies also reported the same results but at a low pH and temperature. For example, Techaoei et al. reported that the best substrates as carbon sources to increase biosurfactant production by *Pseudomonas aeruginosa* SCMU106 were 1% glucose and 0.1% corn oil at pH 7 and 37 °C [45].

Fontes et al. studied the optimization of biosurfactant production by *Yarrowia lipolytica* IMUFRJ 50682 using a factorial design. Maximum biosurfactant production, in a medium containing 10 g of ammonium sulfate, 0.5 g of yeast extract, 4% glucose, and 2% glycerol, using response surface methodology, ΔEI by 110.7%, and ΔST by 108.1%, at pH 7 and 28 °C, has been increased [46].

In contrast to our observations, Gagelidze et al. showed that supplementary carbon sources, such as hexadecane, heptadecane, tetradecane, glycerol, molasses, sunflower, corn and olive oils, at pH 7 and 30 °C did not significantly increase biosurfactant production [47].

Some reports have revealed the important role of bacteria biosurfactants as laundry detergent additives obtained from waste industrial supplements. For instance, Sajna et al. declared that biosurfactants from *Pseudozyma sp.* NII 08165 as an additive for laundry detergents was achieved using 8% waste soybean oil as a carbon source at pH 6 and 30 °C [48]. Bhange et al. reported on biosurfactant and enzyme production from *Bacillus subtilis* PF1 using waste industrial materials. The obtained biosurfactants and enzymes along with other detergent ingredients can enhance stain removal efficiency [7].

Moreover, the time required to achieve maximum production of lipase and biosurfactant decreased.

The obtained results provide a basis for information on the production of lipases and biosurfactants for detergent applications in an identical medium from waste material, which is essential for economically practical production methods. Despite the advantages of lipase and biosurfactant synthesis, production on an industrial scale is still restricted because of the excessive costs involved in the production process. Therefore, further research on cost production of lipase and biosurfactant production by *Ochrobactrum intermedium* strain MZV101 for the detergent industry is required.

## 3. Materials and Methods

### 3.1. Materials

Chemical compounds used in this study include the following: isopropanol (PubChem CID: 3776); *p-*nitrophenyl palmitate (PubChem CID: 73891); sodium deoxycholate (PubChem CID: 23668196); Triton X-100 (PubChem CID: 5590); calcium chloride (PubChem CID: 5284359); dipotassium hydrogen phosphate (PubChem CID: 24450); urea (PubChem CID: 1176). No humans or animals were used in this research.

### 3.2. Microorganism and Growth Medium

In our previous study, the *O. intermedium* strain MZV101 was isolated from the Gheynarje Nir hot spring (latitude 38° 2'8.98"N; longitude 47° 59'0.39"E) in Iran. *O. intermedium* is Gram-negative coccobacilli bacteria that is strictly aerobic, a catalase, and oxidase positive, and their genus belongs to the family Brucellaceae [17]. The 16S rDNA gene sequence of this strain has been recorded in the National Center for Biotechnology Information (NCBI) with accession number KX619441.

The medium for lipase and biosurfactant production by *O. intermedium* strain MZV101 was comprised of the following: 0.2 g/L of MgSO_4_∙7H₂O, 0.01 g/L of FeSO₄∙7H₂O, and 0.01 g/L of CaCl₂. Seven H₂O, 1 g/L of NH_4_Cl, 0.5 g/L of K₂HPO_4_, and 0.1 mL of trace element solution (70 mg/L of ZnCl₂, 100 mg/L of MnCl₂∙4H₂O, 200 mg/L of CoCl₂∙6H₂O, 100 mg/L of NiCl₂∙6H₂O, 20 mg/L of NaMoO₈∙2H₂O, 26 mg/L of Na₂SeO₃∙5H₂O and 1 mL of (25%) HCl) [49]. Flasks containing 100 mL of medium were incubated for 6 days at a 200 rpm agitation speed. Lipase and biosurfactant production by microorganisms was controlled every 24 h.

### 3.3. Lipase Activity Assay (U/mL)

The lipase activity was determined with *p-*NPP as a substrate. For the enzyme solution assay preparation, 10 mL of isopropanol containing 30 mg of *p-*NPP was added to 90 mL of 0.05 M phosphate buffer pH 8 and 207 mg of sodium deoxycholate. To prepare the supernatant, 1 mL of culture was centrifuged by 13300× *g* for 10 min. Thereafter, 0.1 mL of supernatant was added to 2.4 mL of the prepared solution and was incubated for 15 min at 37 °C, and light absorption at a wavelength of 410 nm was measured. Additionally, 0.1 mL of distilled water was added to the solution assay and was considered as a negative control [50].

### 3.4. Emulsification Index (%)

The emulsification index (%) was measured by using 2 mL of petroleum (Persian Gulf Oil Well, Bushehr, Iran) as a substrate supplemented with 2 mL of cell-free supernatant in a test tube, vortexed at a high speed for 2 min, and vertically stirred for 24 h. A 50% emulsification index of the oil layer was defined as good El after 24 h. For the positive control, Triton X-100 was used as a surfactant, and the negative control contained only the buffer and petroleum without surfactant [51]. The formula used to calculate the emulsification index is as follows:

(3) $$EI\;\left(\%\right)=\frac{\mathrm{Height}\;\mathrm{of}\;\mathrm{emulsified}\;\mathrm{layer}\;}{\;\;\mathrm{Total}\;\mathrm{height}\;\mathrm{of}\;\mathrm{the}\;\mathrm{liquid}\;\mathrm{column}}\times 100$$

### 3.5. Statistical Methodology

#### 3.5.1. Plackett–Burman Design

On the basis of the results obtained from the one-factor-at-a-time method, suitable carbon and nitrogen sources were employed for mathematical design. Eleven parameters were investigated in order to achieve maximum lipase and biosurfactant production. The most critical parameters on lipase and biosurfactant production by isolated *O. intermedium* strain MZV101 were studied with Design Expert 10.0.0.6 (Stat Ease Inc., Minneapolis, MN.,USA) using the Plackett–Burman design. Eleven factors including environmental factors (pH and temperature), carbon sources (waste engine oil, waste cocked oil, olive oil and molasses), nitrogen sources (yeast extract and whey), and divalent cations (MgSO₄, CaCl₂ and K₂HPO₄). The factors were studied at pH 8 and 12 and at 20 and 60 °C, and 10 and 30 g/L waste engine oil, 10 and 30 g/L waste cocked oil, 10 and 30 g/L of olive oil, 1 and 5 g/L of molasses, 1 and 5 g/L of yeast extract, 1 and 5 g/L of whey, 0.1 and 0.5 g/L of MgSO₄, 0.8 and 1.2 g/L of CaCl₂, and 0.8 and 1.2 g/L of K₂HPO₄ were used in the defined medium for 6 days. For each variable, high (+) and low (−) levels were tested. Every 12 runs represents the average value of enzyme activity (U/mL), and the emulsification index (%) was taken as a dependent response. The significance of statistical parameters is their interactions, which were determined using ANOVA [14].

#### 3.5.2. Response Surface Methodology

Based on the results of the Plackett–Burman design, significant parameters were screened. For further optimization of lipase and biosurfactant production, significant parameters were studied using response surface methodology (the Box–Behnken design), and data analyzed were examined with Design Expert 10.0.0.6 (Stat Ease Inc., Minneapolis, MN., USA). The experimental design was comprised of 30 runs and five levels. Each experiment was run with two replicates, and the average value of enzyme activity and the emulsification index were represented as a dependent response.

The chosen parameters used in this study are presented in Table 2. The significance of the model was determined by ANOVA. A *p*-value < 0.05 was considered to be significant. The fit of the model was studied using a correlation coefficient (R²), an R-squared coefficient, and an adjusted R-squared coefficient; the results closer to 1 shows a better correlation between experimental and predicted response values. The Adeq Precision measures the signal-to-noise ratio; a ratio greater than 4 is desirable [17].

#### 3.5.3. Validation of the Model

Validation was performed under conditions predicted by the experimental model. The experiments were examined in triplicate at an interval of 24 h. Lipase enzyme activity and the biosurfactant emulsification index was estimated as described earlier. The optimal values for three parameters for enhanced lipase and biosurfactant production were studied experimentally in triplicate.

## Figures and Tables

**Table 1:** The Plackett–Burman design and the results. The table shows 11 independent variables used in the design and shows the obtained results, which can be used to determine the effect of these variables on lipase and biosurfactant production by *O. intermedium* strain MZV101.

Run	pH	Temp. (°C)	Molasses (g/L)	MgSO₄ (g/L)	Waste Engine Oil (g/L)	Waste Cocked Oil (g/L)	K₂HPO₄ (g/L)	Olive Oil (g/L)	CaCl_2_ (g/L)	Whey (g/L)	Yeast Extract (g/L)	Lipase Activity (U/mL)	Emulsification Index (%)
1	8	20	1	0.1	10	10	0.8	10	0.8	1	1	0.571	28.23
2	12	20	1	0.5	30	30	0.8	10	0.8	5	5	3.768	40.54
3	8	60	1	0.1	30	30	1.2	10	1.2	5	1	1.775	33.31
4	8	60	5	0.1	30	10	0.8	30	0.8	5	5	1.341	32.09
5	8	20	5	0.5	10	30	0.8	30	1.2	5	1	0.715	26.48
6	12	60	5	0.1	10	30	0.8	10	1.2	1	5	4.146	43.12
7	12	60	1	0.5	30	10	0.8	30	1.2	1	1	4.984	45.54
8	12	60	5	0.5	10	10	1.2	10	0.8	5	1	4.443	44.02
9	8	60	1	0.5	10	30	1.2	30	0.8	1	5	2.244	34.89
10	12	20	5	0.1	30	30	1.2	30	0.8	1	1	3.211	39.21
11	8	20	5	0.5	30	10	1.2	10	1.2	1	5	0.711	26.43
12	12	20	1	0.1	10	10	1.2	30	1.2	5	5	3.161	41.09

**Table 2:** Results of ANOVA. The table shows the effect of independent variables on lipase and biosurfactant production by *O. intermedium* strain MZV101 using the Plackett–Burman design.

Variable	Lipase Activity (U/mL)						Emulsification Index (%)					
	Sum of Squares	df	Mean Square	*F-*Value	*p*-value		Sum of Squares	df	Mean Square	*F*-Value	*p*-value	
Model	27.04	4	6.76	438.6	<0.0001	significant	525.62	3	175.21	491.47	<0.0001	significant
A-pH	22.29	1	22.29	1446.22	<0.0001		433.08	1	433.08	1214.84	<0.0001	
B-Temp	3.85	1	3.85	249.68	<0.0001		80.03	1	80.03	224.50	<0.0001	
C-Molasses	0.31	1	0.31	20.26	0.0028		12.51	1	12.51	35.08	0.0004	
D-MgSO	0.59	1	0.59	38.25	0.0005		-	-	-	-	-	
Residual	0.11	7	0.015				2.85	8	0.36			
Cor Total	27.15	11					528.47	11				

**Table 3:** The Box–Behnken design and the corresponding response. The experimental design had three independent variables in coded, consisting of 30 runs with three levels, and experiments were carried out in random order.

Run	pH	Temperature (°C)	Molasses (g/L)	Enzyme Activity (U/mL)		Emulsification Index (%)	
				Experimental	Predicted	Experimental	Predicted
1	−1	0	1	4.80	4.81	40.17	40.24
2	−1	1	0	5.99	6.04	43.09	43.04
3	0	0	0	13.01	13.10	81.55	83.17
4	0	1	1	14.53	14.46	89.30	89.08
5	0	0	0	13.15	13.10	83.59	83.17
6	1	1	0	8.60	8.77	68.23	68.25
7	0	1	−1	14.02	13.88	86.74	86.72
8	0	−1	1	11.22	11.38	80.84	80.73
9	−1	−1	0	1.38	1.22	34.21	34.06
10	1	0	1	7.33	7.24	65.24	65.24
11	1	−1	0	6.11	6.06	59.95	59.86
12	0	0	0	13.14	13.10	83.54	83.17
13	1	0	−1	7.35	7.33	63.24	63.04
14	−1	0	−1	2.10	2.19	37.17	37.04
15	0	−1	−1	9.36	9.43	77.62	77.70
16	0	0	0	13.04	13.09	83.59	83.31
17	1	0	1	7.33	7.23	65.24	65.38
18	0	1	−1	14.03	13.87	86.74	86.86
19	−1	0	1	4.80	4.81	40.18	40.38
20	0	1	1	14.53	14.46	89.30	89.22
21	1	0	−1	7.33	7.32	63.24	63.18
22	1	1	0	8.60	8.76	68.23	68.39
23	−1	1	0	5.97	6.03	43.10	43.18
24	0	−1	−1	9.34	9.42	77.63	77.84
25	1	−1	0	6.11	6.05	59.94	60.00
26	−1	0	−1	2.10	2.18	37.17	37.18
27	−1	−1	0	1.38	1.21	34.21	34.20
28	0	0	0	13.11	13.09	83.59	83.31
29	0	−1	1	11.22	11.37	80.85	80.87
30	0	0	0	13.10	13.09	83.59	83.31

**Table 4:** The results of the ANOVA for the response surface quadratic model for lipase and biosurfactant production by *O. intermedium* strain MZV101. The results with a *p*-value less than *p* < 0.0001 were considered as significant.

Source	Lipase		Biosurfactant		
	*F*-value	*p*-value	*F*-value	*p*-value	
Model	5284.42	<0.0001	6371.84	<0.0001	significant
pH	15939.05	<0.0001	14069.85	<0.0001	
Temperature	4966.13	<0.0001	1632.34	<0.0001	
Molasses	285.005	<0.0001	157.30	<0.0001	
AB	209.27	<0.0001	0.96	0.3405	
AC	1.428	0.9702	2.72	0.1157	
BC	2.285	0.9881	1.19	0.2885	
A²	26145.87	<0.0001	40953.20	<0.0001	
B²	9.10	0.0071	0.89	0.3577	
C²	13.58	0.0016	2.21	0.1532	Not significant
Lack of fit	3.22	0.1333	0.07899	0.9999	

Lipase: R² = 0.9998, R-squared = 0.9994 and Adeq Precision = 301.557. Biosurfactant: R² = 0.9997, R-squared = 0.9995 and Adeq Precision = 211.874.

## Supplementary Materials

Supplementary File 1
