# Supplementary material for: Statistical Methodologies for the Optimization of Lipase and Biosurfactant by Ochrobactrum intermedium Strain MZV101 in an Identical Medium for Detergent Applications
Source: Molecules. 2017 Sep 11;22(9):1460. doi: 10.3390/molecules22091460 (PMC6151436; doi:10.3390/molecules22091460)
Supplement: Supplementary File 1 [file molecules-22-01460-s001.pdf]

WWW.NativeEnglishEdit.com

EDITORIAL CERTIFICATE LETTER

This document is to certify that the manuscript listed below was edited for proper English language, grammar, punctuation, spelling, and overall style by one of the highly qualified subject-expert native English speaking editors at **NativeEnglishEdit.com**

The substantive content of the article mentioned below remains the full responsibility of the author/authors:

TITLE OF ARTICLE:

STATISTICAL METHODOLOGY FOR OPTIMIZATION OF LIPASE AND BIOSURFACTANT BY *OCHROBACTRUM INTERMEDIUM* STRAINMZV101 IN THE SAME MEDIA FOR DETERGENT APPLICATION

AUTHOR(S):

MINA ZARINVIARSAGH , GHOLAMHOSSEIN EBRAHIMIPOR , HOSSIEN SADEGHI

REFER CODE:

EE-1396-2224920 ZARIN

Documents receiving this certification should be English-ready for publication; however, the author has the ability to accept or reject our suggestions and changes.
This certificate may be verified at:
Native English Edit
www.birminghamresearchpark.co.uk/tenants/native-english-edit
www.NativeEnglishEdit.com
Birmingham Research Park
Edgbaston
Birmingham B15 2SQ
United Kingdom
